# Exploring the cellulolytic and hemicellulolytic activities of manganese peroxidase for lignocellulose deconstruction

**DOI:** 10.1186/s13068-023-02386-0

**Published:** 2023-09-19

**Authors:** Xiaoqing Liu, Sunjia Ding, Fang Gao, Yaru Wang, Mohammad J. Taherzadeh, Yuan Wang, Xing Qin, Xiaolu Wang, Huiying Luo, Bin Yao, Huoqing Huang, Tao Tu

**Affiliations:** 1 State Key Laboratory of Animal Nutrition and Feeding, Institute of Animal Sciences, Chinese Academy of Agricultural Sciences, Beijing, 100193 China; 2 Biotechnology Research Institute, Chinese Academy of Agricultural Sciences, Beijing, 100081 China; 3 Swedish Centre for Resource Recovery, University of Borås, 50190 Borås, Sweden

**Keywords:** Lignocellulosic biomass, Manganese peroxidase, Cellulose decomposition, Hemicellulose decomposition

## Abstract

**Background:**

A cost-effective pretreatment and saccharification process is a necessary prerequisite for utilizing lignocellulosic biomass (LCB) in biofuel and biomaterials production. Utilizing a multifunctional enzyme with both pretreatment and saccharification functions in a single step for simultaneous biological pretreatment and saccharification process (SPS) will be a green method of low cost and high efficiency. Manganese peroxidase (MnP, EC 1.11.1.13), a well-known lignin-degrading peroxidase, is generally preferred for the biological pretreatment of biomass. However, exploring the role and performance of MnP in LCB conversion will promote the application of MnP for lignocellulose-based biorefineries.

**Results:**

In this study, we explored the ability of an MnP from *Moniliophthora roreri*, *Mr*MnP, in LCB degradation**.** With Mn^2+^ and H_2_O_2_, *Mr*MnP decomposed 5.0 g/L carboxymethyl cellulose to 0.14 mM of reducing sugar with a conversion yield of 5.0 mg/g, including 40 μM cellobiose, 70 μM cellotriose, 20 μM cellotetraose, and 10 μM cellohexaose, and degraded 1.0 g/L mannohexaose to 0.33 μM mannose, 4.08 μM mannotriose, and 4.35 μM mannopentaose. Meanwhile, *Mr*MnP decomposed 5.0 g/L lichenan to 0.85 mM of reducing sugar with a conversion yield of 30.6 mg/g, including 10 μM cellotriose, 20 μM cellotetraose, and 80 μM cellohexose independently of Mn^2+^ and H_2_O_2_. Moreover, the versatility of *Mr*MnP in LCB deconstruction was further verified by decomposing locust bean gum and wheat bran into reducing sugars with a conversion yield of 54.4 mg/g and 29.5 mg/g, respectively, including oligosaccharides such as di- and tri-saccharides. The catalytic mechanism underlying *Mr*MnP degraded lignocellulose was proposed as that with H_2_O_2_, *Mr*MnP oxidizes Mn^2+^ to Mn^3+^. Subsequently, it forms a complex with malonate, facilitating the degradation of CMC and mannohexaose into reducing sugars. Without H_2_O_2_, *Mr*MnP directly oxidizes malonate to hydroperoxyl acetic acid radical to form compound I, which then attacks the glucosidic bond of lichenan.

**Conclusion:**

This study identified a new function of *Mr*MnP in the hydrolysis of cellulose and hemicellulose, suggesting that *Mr*MnP exhibits its versatility in the pretreatment and saccharification of LCB. The results will lead to an in-depth understanding of biocatalytic saccharification and contribute to forming new enzymatic systems for using lignocellulose resources to produce sustainable and economically viable products and the long-term development of biorefinery, thereby increasing the productivity of LCB as a green resource.

## Introduction

With the rising concerns about fossil fuel exhaustion and environmental pollution, it is urgent to explore sustainable green resources for bioenergy production [1]. Lignocellulosic biomass (LCB) is a viable resource for biofuel and biomaterials production due to its low cost, abundance, and often availability as agro-industrial by-products or wastes [2, 3]. It is mainly composed of cellulose, hemicellulose, and lignin rigidly assembled. Each of these three major constituents can be bioconverted to value-added products using a biorefinery approach through biomass conversion consisting of pretreatment, enzymatic hydrolysis, and fermentation [4]. However, the highly complex structure and rigid recalcitrant nature of LCB is the main barrier to effectively converting LCB to bio-products [5]. To process LCB into biofuel, pretreatment is needed to efficiently remove lignin, making cellulose and hemicelluloses exposed for enzymatic hydrolysis [6, 7]. Furthermore, several classes of enzymes are required to completely convert LCB into fermentable sugars [8]. Therefore, a cost-effective pretreatment and saccharification process are prerequisites for utilizing the LCB in biofuel production.

In contrast to conventional physical and chemical pretreatment methods, biological pretreatment using ligninolytic enzymes (laccase, manganese peroxidase, lignin peroxidase, and versatile peroxidase) is a greener and cleaner method due to its higher safety, milder process conditions, and higher reaction specificity [9]. Combining the biological pretreatment with the subsequent saccharification steps will be a lower energy and less time process than sequential steps, which can be achieved by mixing ligninolytic enzymes with cellulolytic enzymes in a single step for simultaneous biological pretreatment and saccharification process (SPS) [10]. Although several successful enzymatic SPS methods have been reported [11–13], poor efficiency and higher production cost make the enzymatic SPS process unpopular. These issues can be solved by utilizing more robust and multifunctional enzyme systems. Except for laccase, other ligninolytic enzymes have seldom been reported to be used for delignification in SPS [14, 15]. While the conventional cellulolytic enzymes for saccharification are glycosidic hydrolases. However, the recently identified lytic polysaccharide monooxygenases (LPMOs) can cleave recalcitrant polysaccharides by oxidation [16–19], revolutionizing the understanding of enzyme-based saccharification. Given that ligninolytic enzymes are also oxidoreductases, it is worth exploring the cellulose degradation ability of ligninolytic enzymes, which can significantly improve the SPS efficiency for biomass conversion.

Manganese peroxidase (MnP, EC 1.11.1.13), a well-known lignin-degrading peroxidase, can oxidatively depolymerize lignin in an H_2_O_2_-assisted reaction by oxidizing Mn^2+^ to Mn^3+^, which is subsequently chelated by organic acids forming a diffusible oxidant to degrade lignin [20, 21], aromatic compounds [22, 23], pollutants and dyes [24–26]. MnPs are promising biocatalysts for converting lignin-based feedstock into high-value products, such as bioethanol and others, through lignin deconstruction/delignification [27]. However, their role in cellulose decomposition has not been thoroughly studied yet. Exploring the role and performance of MnP in LCB conversion will facilitate the utilization of MnP for lignocellulose-based biorefineries.

Most studies of MnPs focus on their ability to biodegrade organic pollutants, toxins, etc. This study explored its ability in LCB decomposition by using an MnP from *Moniliophthora roreri*, *Mr*MnP, distinguished from other enzyme counterparts by its high-level secretory expression with a strong potential application prospect [28, 29]. The effects of buffer components, pH, and H_2_O_2_ on *Mr*MnP activity were first examined. Then the optimal reaction conditions were performed to examine its ability in cellulose (CMC and lichenan) and hemicellulose (xylan and mannan) degradation. It was found that the MnP-driven Mn^3+^-malonate complex hydrolyzed CMC and Mannohexaose to reducing sugars, and *Mr*MnP decomposed lichenan independently of Mn^2+^ and H_2_O_2_. The versatility of *Mr*MnP in LCB deconstruction was further verified by decomposing locust bean gum and wheat bran into oligosaccharides such as di- and tri-saccharides. This study demonstrated previously unknown cellulolytic and hemicellulolytic activities of *Mr*MnP. This new function of *Mr*MnP in the hydrolysis of cellulose and hemicellulose, coupled with its known delignification activity, makes this enzyme a versatile enzyme for SPS of lignocellulosic biomass. The results will lead to an in-depth understanding of biocatalytic saccharification and contribute to forming new enzymatic systems for producing environmentally friendly products from lignocellulose and the long-term development of biorefinery.

## Results and discussion

### Characterization of the *Mr*MnP

**Fig. 1:**
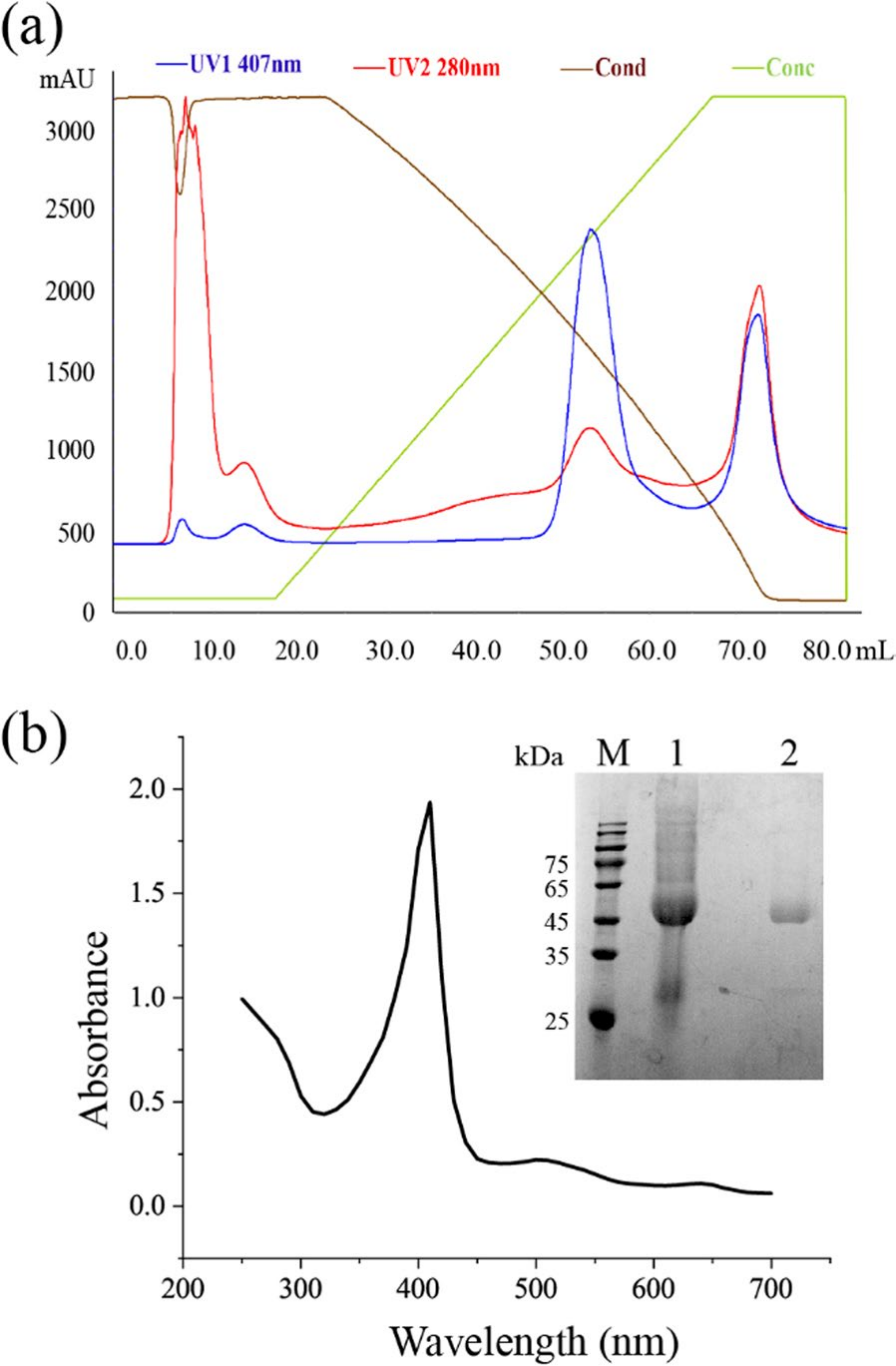
Purification of *Mr*MnP. **a** FPLC elution profile of the purification step of *Mr*MnP performed on a HiTrapTM Phenyl HP FPLC column. Heme absorption at 407 nm (blue line), total protein at 280 nm (red line), (NH_4_)_2_SO_4_ concentration (brown line), percentage of 10 mM McIlvaine buffer (green line). **b** UV–Vis absorption spectra of *Mr*MnP. The inset shows SDS-PAGE analysis of purified *Mr*MnP. Lane M: protein ladder; lane 1: crude extract showing expressed *Mr*MnP; lane 2: purified *Mr*MnP (~ 45 kDa)

*Mr*MnP can be heterologously expressed in *Pichia pastoris* at a high level (132 mg/L) [28]. In this study, the *Mr*MnP, expressed in *P*. *pastoris* as before [29], was purified using hydrophobic-interaction chromatography (Fig. 1a). SDS-PAGE revealed that the purified *Mr*MnP had a single band with a molecular mass of about 45 kDa (Fig. 1b), which was slightly higher than the theoretical molecular weight of 39.5 kDa. This is most likely due to post-translational glycosylation. UV–visible absorption spectrum scanning analyses showed an absorbance peak at 408, indicating the correct heme incorporation (Fig. 1b). The *R*_Z_ (A_408_/A_280_) value was about 2.4, indicating the high purity of the enzyme, which was consistent with SDS-PAGE results. *Mr*MnP can oxidize phenolic substrate DMP with the specific activity of 4.2 ± 0.2 U/mg, as well as ABTS (34.9 ± 4.4 U/mg) and MnSO_4_ (36.2 ± 5.1 U/mg), exhibiting the characteristics of short MnPs which can oxidate low-redox-potential substrates (ABTS, 2,6-DMP) [28].

### Effects of buffer components on *Mr*MnP activity

**Fig. 2:**
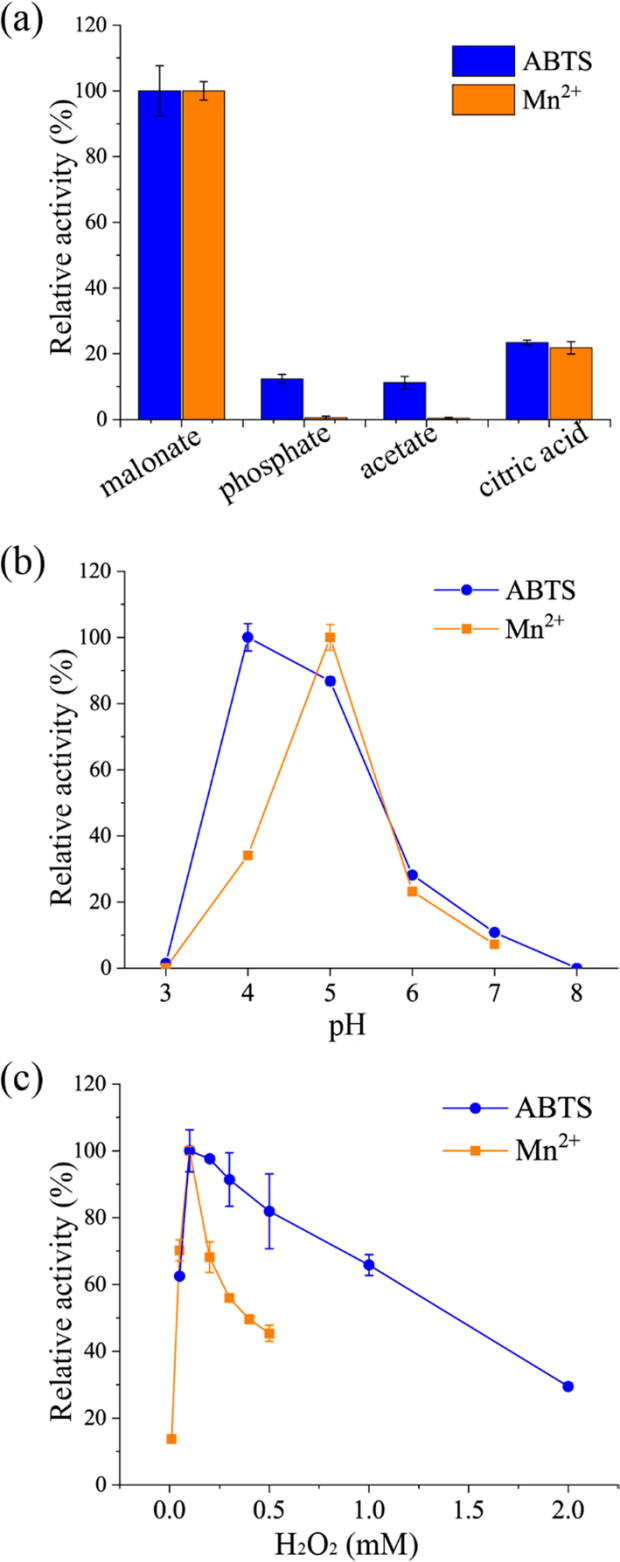
The effect of buffer components (**a**), pH (**b**), and H_2_O_2_ concentration (**c**) on oxidation of ABTS and Mn^2+^ by *Mr*MnP. Error bars represent standard deviation calculated based on triplicate experiments

Due to the importance of organic acids (the enzymatically generated Mn^3+^ chelator) in MnP-catalyzed oxidation [30], four different buffer solutions (malonate buffer, citric acid buffer, phosphate buffer, acetate buffer, 50 mM, pH 5) were selected to investigate the effects of buffer components on *Mr*MnP activity (Fig. 2a). For both substrates ABTS and MnSO_4_, the highest activity was obtained in malonate buffer, which is in agreement with the previous study that malonate is the most effective chelator [31]. Unlike ABTS, *Mr*MnP could not oxidize Mn^2+^ to Mn^3+^ in phosphate buffer and acetic acid–sodium acetate buffer. No Mn^3+^ was detected in the phosphate buffer, confirming that C2 and C3 dicarboxylic or a-hydroxyl acids are needed to stimulate the MnP activity [32]. Besides, the Mn^2+^ was not oxidized to Mn^3+^ in the acetate buffer, probably because H_2_O_2_ may reduce the resulting Mn3 + -acetate complex without a phenolic terminal substrate [33]. The oxidation rate of Mn^2+^ by MnP was also extremely slow in the reaction system containing acetate [34]. Thus, malonate buffer is used for all the following reactions unless otherwise specified.

### Effects of pH and H_2_O_2_ on *Mr*MnP activity

The pH–activity profile was significantly narrower, consistent with the data for most reported fungal MnP [26]. For the ABTS oxidation activity, it exhibited a maximum at pH 4 and retained more than 80% of its maximum activity between pH 4 and 5, but it was completely lost at pH 3 and 8 (Fig. 2b); For the Mn^2+^ oxidation activity, it exhibited a maximum at pH 5 and was completely lost at pH 3. It was reduced to 34%, 23%, and 7% at pH 4, 6, and 7, respectively (Fig. 2b). Considering that MnP functions through oxidizing Mn^2+^ and *Mr*MnP is most stable at pH 5 [28], the following reactions were conducted at pH 5.

As an essential factor in initiating the MnP catalytic cycle, the concentration of H_2_O_2_ also affected the activity of *Mr*MnP (Fig. 2c). When the concentration of H_2_O_2_ was 2 mM, the residual ABTS oxidation activity was less than 40%. This may be because excessive H_2_O_2_ can convert MnP into MnP compound III [35], a superoxide anion (O_2_·^−^) having Fe^3+^ species, which cannot participate in normal substrate oxidation reactions [36]. The enzyme retained > 60% ABTS oxidation activity as H_2_O_2_ concentration was 0.05 ~ 1 mM, especially 0.1 mM. The effect of H_2_O_2_ concentration on the oxidation activity of MnSO_4_ was greater than that of ABTS. The optimum concentration for Mn^2+^ oxidation activity was 0.1 mM, and the activity decreased by more than 30% when the concentration was increased to 0.2 mM. When the concentration was greater than or equal to 0.4 mM, the activity decreased to less than half. Thus, the optimum concentration for Mn^2+^ oxidization, 0.1 mM, was used for the following reactions.

### *Mr*MnP catalyzes the degradation of cellulose

**Fig. 3:**
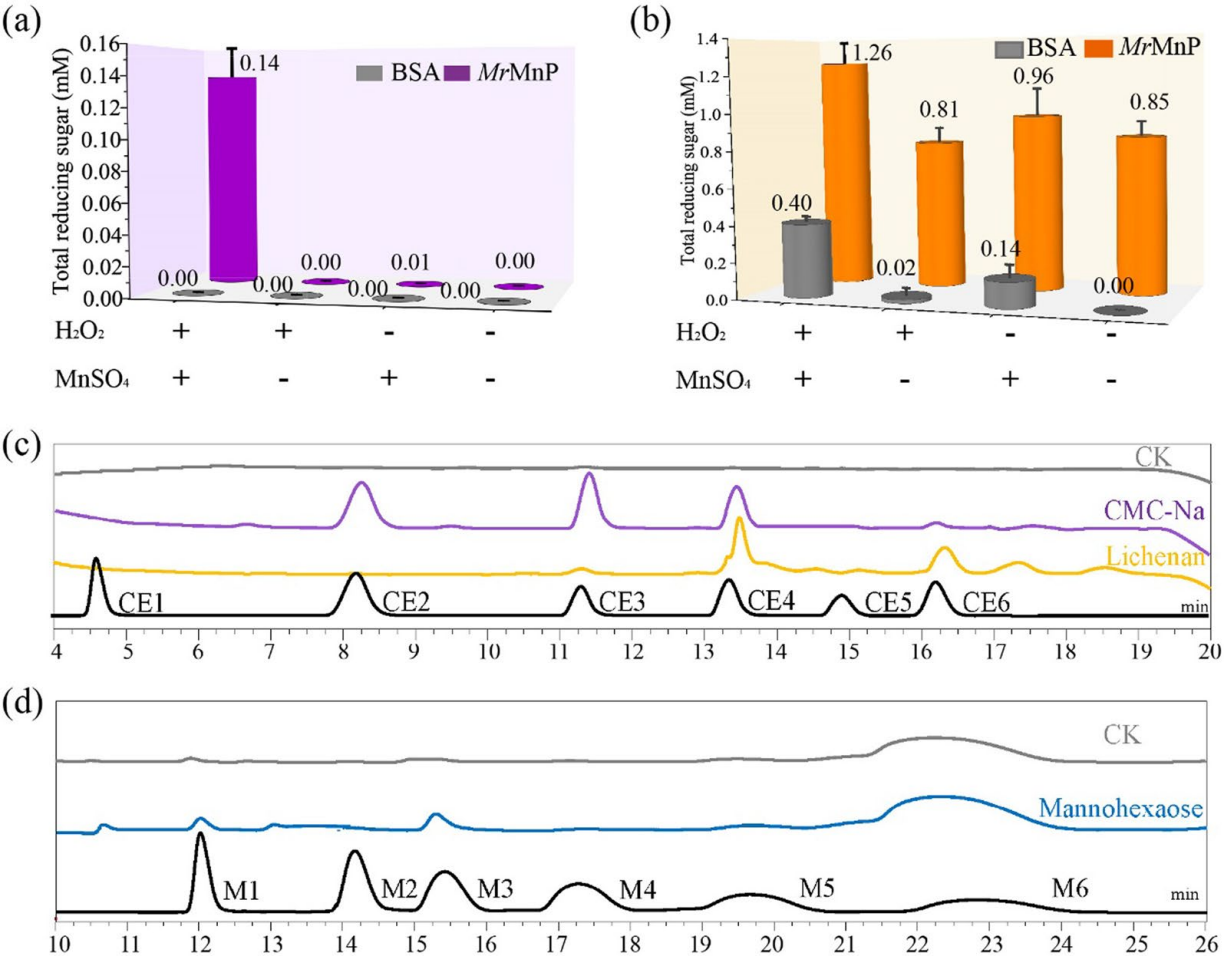
Analysis of the degradation products from CMC, lichenan, and mannohexaose. **a** Reducing sugar from CMC (0.5% (m/V)). **b** Reducing sugar from lichenan (0.5% (m/V)). **c** HPAEC analysis of hydrolysates of CMC and lichenan. **d** HPAEC analysis of hydrolysates of mannohexaose (1.0 g/L). The reaction was conducted in 50 mM malonate buffer (pH 5) containing 2.5 mg *Mr*MnP, 1.0 mM MnSO_4_, and 0.1 mM H_2_O_2_ at 37 °C for 24 h. Error bars represent standard deviation calculated based on triplicate experiments. CE1: glucose, CE2: cellobiose, CE3: cellotriose, CE4: cellotetraose, CE5: cellopentaose, CE6: cellohexaose, M1: mannose, M2: mannobiose, M3: mannotriose, M4: mannotetraose, M5: mannopentaose, M6: mannohexaose

To examine whether *Mr*MnP can catalyze cellulose decomposition, CMC and lichenan were used as cellulosic substrates. As seen in Fig. 3a, 0.14 mM of reducing sugar was released from CMC after treatment by *Mr*MnP with MnSO_4_ and H_2_O_2_ in malonate buffer (50 mM, pH 5) at 37 °C for 24 h. The reducing sugar conversion yield from CMC was 5.0 mg/g. *Mr*MnP alone or *Mr*MnP and H_2_O_2_ had no cellulolytic activity. MnP from *P*. *chrysosporium* (*Pc*MnP) was first reported to produce reducing sugar from CMC in 50 mM acetate buffer (pH 4.5). However, the product's composition was not analyzed in detail [37]. We further analyzed the products using HPAEC-PAD. The results showed that 0.04 mM CE2, 0.07 mM CE3, 0.02 mM CE4, and 0.01 mM CE6 were produced, a total of 0.13 mM, which was lower than the reducing sugars by DNS method, indicating that some polysaccharides, which were not detected by HPAEC-PAD, were still present (Fig. 3c).

MrMnP also degraded lichenan but was different from CMC (Fig. 3b). *Mr*MnP itself shocked us by producing 0.85 mM of reducing sugar from lichenan with a conversion yield of 30.6 mg/g. The control group containing the same amount of BSA did not detect any reducing sugar, suggesting that this resulted from *Mr*MnP rather than the self-degradation of lichenan. After adding MnSO4, the increment of reducing sugar in the experimental and control groups was similar (0.11 and 0.14 mM, respectively). Similarly, adding H_2_O_2_ and MnSO_4_ simultaneously increased the reducing sugar by 0.41 mM in the experimental group and 0.40 mM in the control group, respectively. The HPAEC-PAD result showed that the degradation product by *Mr*MnP contained 0.01 mM CE3, 0.02 mM CE4, and 0.08 mM CE6 (Fig. 3c), a total of 0.11 mM, indicating that the polymerization degree of the product is very high, which is not detected by HPAEC-PAD. Overall, these results suggest that MnP decomposed lichenan independently of Mn^2+^ and H_2_O_2_, which differs from CMC's.

### *Mr*MnP catalyzes the degradation of hemicellulose

To examine whether *Mr*MnP can catalyze hemicellulose decomposition, xylan and mannan, two important hemicellulose components, were used as hemicellulosic substrates. Although the amount of substrate was reduced, no reducing sugar was detected in the reaction products. Interestingly, when the concentration of *Mr*MnP was adjusted from 2.5 mg/mL to 25 μg/mL, it produced 0.33 μM M1, 4.08 μM M3, and 4.35 μM M5 in 24 h from mannohexaose with H_2_O_2_ and MnSO_4_ (Fig. 3d). A possible explanation for this might be that high concentrations of *Mr*MnP have higher oxidation activity, which may directly oxidize substrates into other non-reducing sugar products. *Mr*MnP could not decompose xylan; on the one hand, it might be because it had very low xylanase activity as *Pc*MnP reported by Min et al. [37]; on the other hand, it might be because the reaction condition was not suitable as Min et al. found that the optimal temperature and pH for xylan decompose by *Pc*MnP was different from that of Mn^2+^ oxidation [37]. Since the hydrolysis products can only be formed by adding H_2_O_2_ and MnSO_4_, *Mr*MnP may degrade mannohexaose and CMC similarly.

### *Mr*MnP catalyzes the degradation of the raw material substrate

**Fig. 4:**
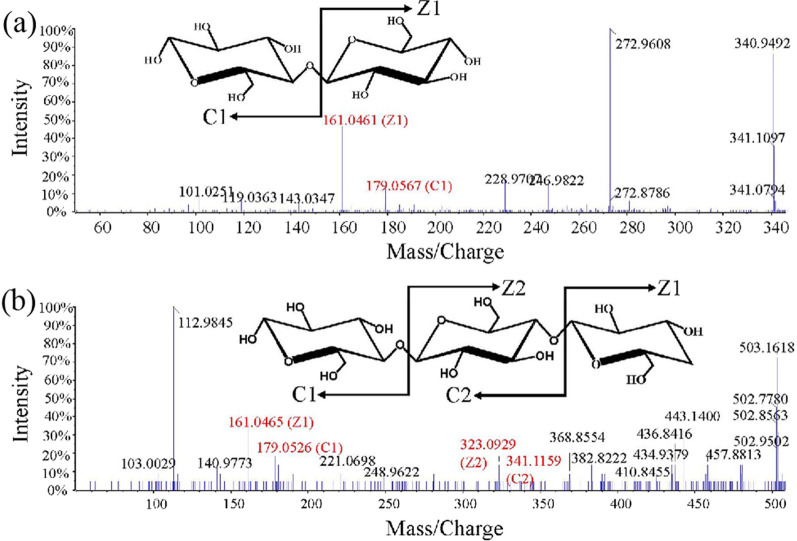
Mass spectra of the degradation products from wheat bran and locust bean gum. **a** Disaccharide released from wheat bran and locust bean gum (0.5% (m/V)). **b** Trisaccharide released from locust bean gum (0.5% (m/V)). The reaction was conducted in 50 mM malonate buffer (pH 5) containing 2.5 mg *Mr*MnP, 1.0 mM MnSO_4_, and 0.1 mM H_2_O_2_ at 37 °C for 24 h

Given the ability of (hemi)cellulose degradation, we evaluated whether *Mr*MnP can decompose LCB using the raw material wheat bran and locust bean gum as substrates. In the presence of MnSO_4_ and H_2_O_2_, 1.51 and 0.82 mM of reducing sugars were released from wheat bran and locust bean gum after treatment with *Mr*MnP at 37 °C for 24 h, respectively. The reducing sugar conversion yield from wheat bran and locust bean was 54.4 mg/g and 29.5 mg/g, respectively. The degradation products were further analyzed using UHPLC–HRMS in negative ion mode. In the wheat bran degradation product, peaks with mass-to-charge ratio (m/z) of 340.94 and 342.96 were visible, and this substance may be a disaccharide composed of two hexose units. To further determine the structure of this substance, fragment patterns were further analyzed. As shown in Fig. 4a, the fragment ions C1 (m/z, 179.05) and Z1 (m/z, 161.04) combined precisely to form intact disaccharides. The degradation products of locust bean gum also contain disaccharides. Moreover, trisaccharide was also present. The fragment ions C1 (m/z, 179.05), C2 (m/z, 341.11), Z1 (m/z, 161.04), and Z2 (m/z, 323.09) are the characteristic fragments of trisaccharide (Fig. 4b). These results indicated that *Mr*MnP could hydrolyze raw material substrates to reducing sugars. As *Mr*MnP has cellulolytic, hemicellulolytic, and delignification activities, it has excellent potential for SPS of lignocellulosic biomass in biorefinery.

### Proposed catalytic mechanism

**Fig. 5:**
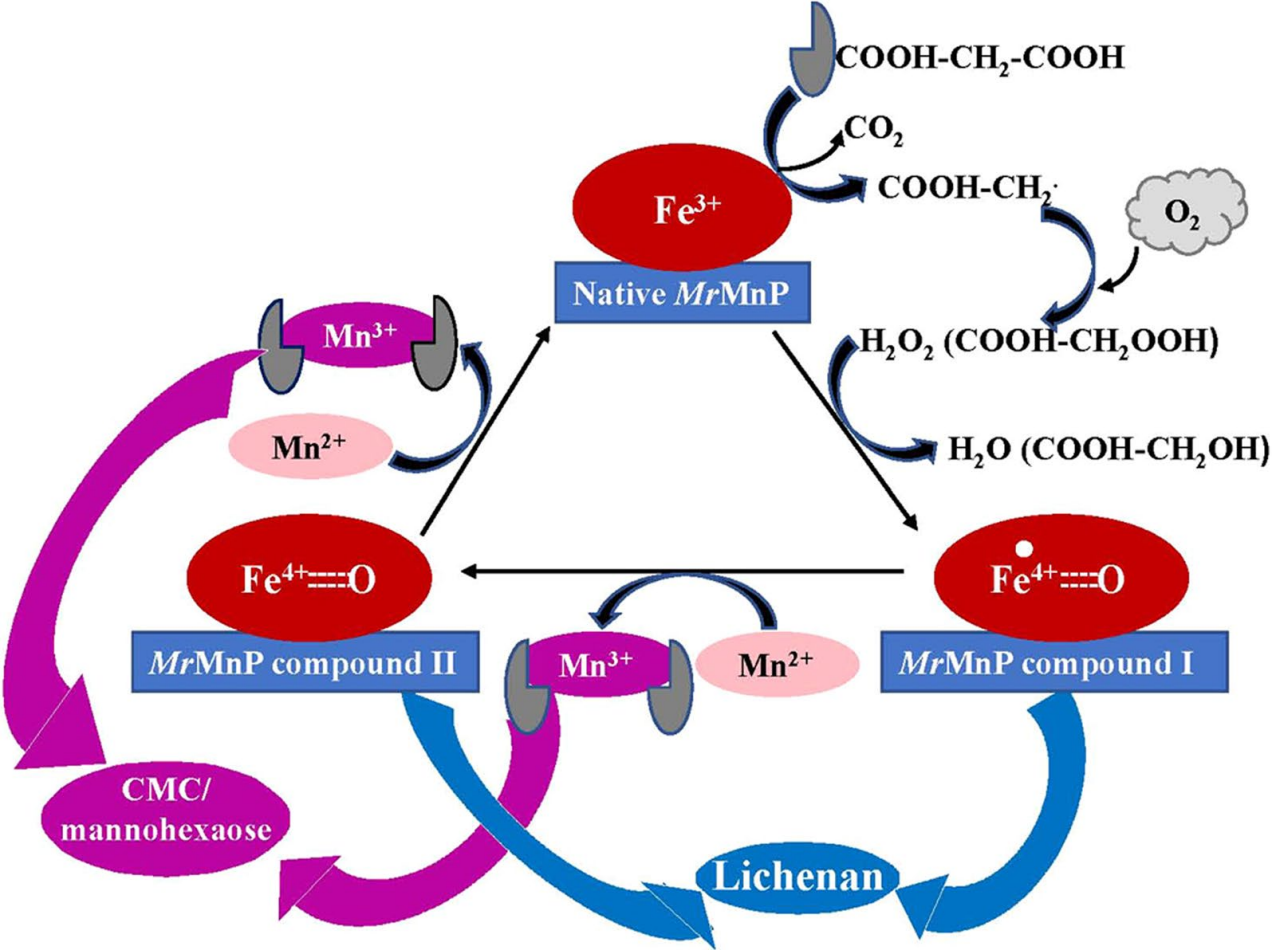
Proposed schematic for the catalytic mechanism underlying *Mr*MnP degrade CMC, lichenan, and mannohexaose

The detection of monosaccharides and oligosaccharides in the CMC, lichen, and mannohexaose degraded products formed by *Mr*MnP suggested that *Mr*MnP has cellulolytic and hemicellulolytic activity, which is different from LPMO catalyzing oxidative cleavage of glycosidic bonds [19, 38]. H_2_O_2_ and Mn^2+^ were required for the degradation of CMC and mannohexaose, as well as lignin. In addition, MnP from *P. chrysosporium* was found to release peroxidized glucose and glucose from cellobiose [37]. Thus, we proposed that the catalytic mechanism underlying *Mr*MnP decomposed CMC and mannohexaose is the normal peroxidase catalytic cycle [35], wherein the native MnP is oxidized by H_2_O_2_ in a two-electron transfer step to form reactive intermediate MnP Compound I (Fe^4+^ oxo-porphyrin radical cation), and the native MnP is recovered through reducing the compound I with Mn^2+^ in two single one-electron transfer steps with the intermediate formation of MnP Compound II. The generated Mn^3+^, chelated with an organic acid such as malonate, then degraded CMC and mannohexaose into reducing sugars (Fig. 5).

However, the degradation of lichen polysaccharides catalyzed by *Mr*MnP was independent of H_2_O_2_ and Mn^2+^, completely different from ordinary MnP's mechanism. *Mr*MnP was regarded as a short MnP due to its 343 amino acid length and ability to oxidize low redox potential [28, 39]. The short MnPs are capable of directly oxidizing low-redox-potential compounds such as phenols, amines, and small dye compounds, without Mn^2+^, through an additional active site containing an exposed heme edge to indirect contact with the δ-position of the porphyrin macrocycle by compound I and II [40]. Furthermore, it has been reported that MnP can oxidize the organic acid to stimulate MnP activity without H_2_O_2_ [41, 42]. Thus, we proposed that *Mr*MnP can directly oxidize malonate to hydroperoxyl acetic acid radical (COOH–CH2OO^**.**^), which is transformed to a hydroperoxide (COOH–CH2OOH) using by *Mr*MnP to form compound I [42]. The *Mr*MnP compound I then attack the glucosidic bond of lichenan. To further elucidate the proposed mechanism experimentally, isotope-labeling experiments are required in future studies.

## Conclusion

As cost-effective pretreatment and saccharification are vital steps for the LCB convention, a multifunctional enzyme that can function in both pretreatment and saccharification phases will significantly improve the efficiency of lignocellulosic-based biorefinery. In this study, we report *Mr*MnP's unknown cellulolytic and hemicellulolytic activity. Being a lignin-degrading enzyme, MnP can hydrolyze CMC, Mannohexaose, and lichenan to reducing sugars, suggesting its versatility in LCB decomposition. This was verified by decomposing locust bean gum and wheat bran into oligosaccharides such as di- and tri-saccharides. With its lignin degradation properties, *Mr*MnP is a super enzyme with great potential for lignocellulosic degradation. It would contribute to forming an economic cellulolytic cocktail for producing sustainable and economically viable products from lignocellulose and the long-term development of biorefinery.

## Materials and methods

### Strains and chemicals

The *P*. *pastoris* X33 transformant bearing the *Mr*MnP manganese peroxidase gene from *Moniliophthora roreri* (GenBank accession number: ESK95360.1) was constructed by our lab [29], and this construct was used for *Mr*MnP production. The substrates carboxymethyl cellulose (CMC), locust bean gum, 2,2’-azinobis(3-ethylbenzothiazoline-6-sulfonic acid) (ABTS), 2,6-dimethylphenol (2,6-DMP), and the standard substances glucose (CE1), cellobiose (CE2) and mannose (M1) were purchased from Sigma-Aldrich (St. Louis, MO). The substrates lichenan, mannohexaose, and the standard substances cellotriose (CE3), cellotetraose (CE4), cellopentaose (CE5), cellohexaose (CE6), mannobiose (M2), mannotriose (M3), mannotetraose (M4), mannopentaose (M5) and mannohexaose (M6) were purchased from Megazyme (Bray, Wicklow, Ireland). The other chemicals used in this research are of analytical grade and commercially available.

### Enzyme expression and purification

*Mr*MnP was produced in a 6-L fed-batch fermentation process as described before [29]. The fermentation supernatant, which was concentrated by a 10-kDa ultrafiltration membrane, was purified using a HiTrapTM Phenyl HP FPLC column (GE Healthcare, Uppsala, Sweden), followed by a RESOURCETM Q (6 mL) FPLC column (GE Healthcare) as described previously [43]. The enzyme concentration was determined by the Bradford assay. The purified *Mr*Mnp were analyzed by sodium dodecyl sulfate–polyacrylamide gel electrophoresis (SDS-PAGE) using a 12% polyacrylamide gel and scanned by UV–visible absorption spectrum with a microplate reader in the wavelength range of 250 − 700 nm. The protein purity was evaluated by calculating the *R*_Z_ value, where *R*_Z_ = A_407_/A_280_.

### Biochemical characterization

*Mr*MnP activity was determined spectrophotometrically by monitoring the oxidation of 1.0 mM ABTS (*ϵ*_420_ = 36,000 /M/cm), 2,6-DMP (ϵ_468_ = 49,600 /M/cm), and MnSO_4_ (*ϵ*_270_ = 11,590 /M/cm) at 420, 468, and 270 nm, respectively, using UV Vis spectrophotometer (Hitachi, model 8543). Reactions were performed in 200 μL of 50 mM malonate buffer containing 5 μg/mL *Mr*MnP and 1 mM MnSO_4._ The Reactions were initiated by the addition of 0.1 mM H_2_O_2_. The data were recorded every 30 s for 3 min at 30 °C. One unit (U) of enzyme activity was defined as the amount of enzyme oxidizing 1 μmol substrate or producing 1 μmol oxidation product per minute under the assay conditions. Optimum conditions for oxidation of ABTS and MnSO_4_ were determined. To determine optimum pH and H_2_O_2_ concentration, 50 mM malonate buffer (pH 3.0 − pH 7.0) and H_2_O_2_ (0.01 − 2 mM) were used. To determine the buffer, the pH and H_2_O_2_ concentration are maintained at the determined optimum.

### Enzymatic hydrolysis of (hemi)cellulosic substrates and raw material substrate

To investigate the (hemi)cellulosic decomposing ability of *Mr*MnP, various (hemi)cellulosic substrates (5.0 g/L of CMC, lichenan, and 1.0 g/L of mannohexaose) were reacted with 2.5 mg *Mr*MnP in 1 mL malonate buffer (50 mM, pH 5) containing 1.0 mM MnSO_4_, and 0.1 mM H_2_O_2_ at 37 °C for 24 h, respectively. To investigate the lignocellulose decomposing ability of *Mr*MnP, the locust bean gum and sulfuric acid pretreated (2% H_2_SO_4_, 121 °C, 1 h, the ratio of straw to liquid 10%) wheat bran were used as raw material substrate. The reaction system and condition were consistent with (hemi)cellulose substrates. The BSA was served as the control.

### Hydrolysates analysis

The total reducing sugar liberated from enzymatic hydrolysis was measured using the 3,5-dinitrosalicylic acid (DNS) assay [44] with glucose as the standard. The hydrolysates of (hemi)cellulosic substrates were separated and quantified using high-performance anion-exchange chromatography with pulsed amperometric detection (HPAEC-PAD; ThermoFisher, Sunnyvale, CA) equipped with a CarboPac™ PA-100 (4 mm × 250 mm) column. A multi-step gradient was performed to analyze the hydrolysates of CMC and lichenan using the previous method [45]. The substances CE1, CE2, CE3, CE4, CE5, and CE6 were used as the standards. To analyze the hydrolysates of mannohexaose, the eluents were deionized water (eluent A) and 0.1 M sodium hydroxide (eluent B) at a flow rate of 0.45 mL/min, using the multi-step procedure as follows: 0–4 min, isocratic, 20% B; 4–5 min, linear, 20%–100% B; 5–25 min, isocratic, 100% B; 25–28 min, linear, 100%–20% B; and 28–31 min, isocratic, 20% B. The substances M1, M2, M3, M4, M5, and M6 were used as the standards. The hydrolysates of raw material substrate were analyzed by ultra-high-performance liquid chromatography–high-resolution mass spectrometry (UHPLC–HRMS; TripleTOF™ 5600 + , AB SCIEX, USA) with Poroshell 120 EC-C8 (100 mm × 4.6 mm, 4 μm, Agilent) as described previously [45].

## Data Availability

All data generated or analyzed during this study are included in this manuscript.

## Abbreviations

LCB: Lignocellulosic biomass
SPS: Simultaneous biological pretreatment and saccharification process
LPMOs: Lytic polysaccharide monooxygenases
CMC: Carboxymethyl cellulose
ABTS: 2,2’-Azinobis(3-ethylbenzothiazoline-6-sulfonic acid)
2,6-DMP: 2,6-Dimethylphenol
CE1: Glucose
CE2: Cellobiose
CE3: Cellotriose
CE4: Cellotetraose
CE5: Cellopentaose
CE6: Cellohexaose
M1: Mannose
M2: Mannobiose
M3: Mannotriose
M4: Mannotetraose
M5: Mannopentaose
M6: Mannohexaose
SDS-PAGE: Sodium dodecyl sulfate–polyacrylamide gel electrophoresis
DNS: 3,5-Dinitrosalicylic acid
HPAEC-PAD: High-performance anion-exchange chromatography with pulsed amperometric detection
UHPLC–HRMS: Ultra-high-performance liquid chromatography–high-resolution mass spectrometry
